# A Chitosan-Binding Protein Mediated the Affinity Immobilization of Enzymes on Various Polysaccharide Microspheres

**DOI:** 10.3390/foods14111981

**Published:** 2025-06-04

**Authors:** Dexin Zhao, Shiguo Peng, Feifei Chen, Alei Zhang, Kequan Chen

**Affiliations:** State Key Laboratory of Materials-Oriented Chemical Engineering, College of Biotechnology and Pharmaceutical Engineering, Nanjing Tech University, Nanjing 211816, China; zhaodexin2018@163.com (D.Z.); 18299288661@163.com (S.P.); feifeichen@njtech.edu.cn (F.C.)

**Keywords:** enzyme catalysis, simultaneous purification and immobilization, chitosan-binding protein, polysaccharide microspheres, continuous catalysis

## Abstract

In this study, we developed an innovative method for one-step enzyme purification and immobilization utilizing polysaccharide-based microspheres through a chitosan-binding module that mediated affinity adsorption. The chitosan-binding domain derived from *Paenibacillus* sp. IK-5 was genetically fused with multiple target enzymes (lysine decarboxylase, glutamate oxidase, and formate dehydrogenase), all of which were successfully expressed in soluble forms. Three distinct polysaccharide microspheres with optimized surface characteristics were engineered to facilitate the concurrent purification and immobilization of these fusion enzymes. Comprehensive characterization using organic elemental analysis, fluorescence microscopy, and thermogravimetric analysis confirmed the efficient immobilization of fusion enzymes. Remarkably, the immobilized enzymes demonstrated exceptional operational stability, maintaining over 80% of their initial catalytic activity after ten consecutive reuse cycles. This study establishes a robust and versatile platform for enzyme immobilization, providing significant advantages in biocatalyst engineering applications.

## 1. Introduction

Enzymes, which act as highly efficient and substrate-specific biocatalysts, are widely used in food, pharmaceutical, and chemical industries [1,2,3]. Nevertheless, the widespread implementation of enzymatic systems in food industrial applications is often limited by their substantial economic constraints, primarily stemming from challenges associated with operational stability and recyclability [4,5]. Enzyme immobilization has emerged as a promising strategy for significantly improving both the operational stability and reusability of biocatalysts [6,7]. Enzyme immobilization can be classified into two primary categories: irreversible methods relying on covalent bonding (e.g., cross-linking and covalent binding) and reversible strategies involving non-covalent interactions, such as entrapment and adsorption techniques [8,9]. However, enzyme immobilization methodologies typically require two distinct sequential stages: purification followed by immobilization. These processes are not only technically complex and economically demanding but also frequently lead to substantial enzyme activity loss. Furthermore, conventional enzyme immobilization methods often involve toxic substances, limiting their suitability for food and pharmaceutical applications. Therefore, the development of novel immobilization strategies that combine high efficiency with harmlessness represents a critical research imperative in biocatalysis.

Simultaneous purification and immobilization represent an integrated biocatalytic engineering approach that consolidates the conventional two-step process of independent purification followed by immobilization into a single operational procedure, thereby significantly streamlining enzyme processing. This innovative methodology has been experimentally validated to significantly enhance process efficiency, substantially reduce operational costs, and effectively preserve enzymatic activity and structural stability, which has gained significant traction in protein purification, particularly through affinity tags [10,11,12]. For instance, Zhou et al. immobilized His-tagged glucose dehydrogenase on NiFe_2_O_4_ magnetic nanoparticles, achieving enhanced pH/thermal stability and retaining over 65% activity after ten cycles [13]. Abdelhamid et al. utilized a Silaffin-3-derived pentalysine cluster as a novel fusion tag for the immobilization of recombinant catalase onto silica substrates. This approach enabled efficient enzyme recovery, achieving 85% yield while maintaining 91% purity, demonstrating the effectiveness of the proposed methodology [14]. However, these protocols present several inherent limitations, particularly concerning the intricate fabrication processes required for carrier materials, substantial economic constraints, and significant compromise in enzymatic activity retention.

In contrast to conventional affinity tags, carbohydrate-binding modules (CBMs) derived from glycoside hydrolases exhibit unique advantages through their ability to form specific non-covalent interactions with natural polysaccharide matrices [15,16,17]. At present, all CBMs can be classified into 106 families according to the carbohydrate-active enzyme database based on amino acid sequence, binding specificity, and structure [18]. CBMs are important components of carbohydrate-active enzymes, which are widely found in bacteria, fungi, animals, and plants. The CBM-mediated immobilization of the enzyme is notable for the significant advantage that the carriers themselves offer, in that they are renewable, non-toxic, and biocompatible. In addition, the strategy allows for the selective purification and immobilization of proteins fused with CBMs [19]. In view of the aforementioned advantages, there has been an increasing focus on simultaneous purification and immobilization strategies that utilize polysaccharides and CBM-fused enzymes. For instance, Hu et al. successfully immobilized β-glucosidase fused with cellulose-binding module 3 (CBM3) onto regenerated amorphous cellulose matrices, and the immobilized biocatalyst maintained the capacity to hydrolyze 96.9% of daidzin and 98.9% of genistin after the 30th reuse [20]. Qin et al. demonstrated the successful immobilization of a chitosanase fused with a carbohydrate-binding module from family 56 (CBM56) onto curdlan matrices, achieving a remarkable adsorption capacity of 50.75 mg/g [21]. In our previous study, chitin material was employed to immobilize L-lysine decarboxylase CadA that was fused with a chitin-binding domain (ChBD) via simultaneous purification and immobilization, demonstrating an efficient bioconversion of L-lysine to cadaverine with excellent operational stability [22]. However, currently reported CBMs are characterized by their high substrate specificity, displaying selective affinity toward singular polysaccharide matrices, thus limiting their universal applicability in biocatalytic processes.

In this study, we characterized a novel chitosan-binding module (CBM) derived from the C-terminus of a chitosanase of *Paenibacillus* sp. IK-5, which exhibits unique polysaccharide-binding versatility towards chitin, chitosan, and cellulose substrates. The CBM consists of modules with two identical β-cores and helical structures. Through the implementation of sequence similarity analysis, it has been determined that each module exhibits an average sequence similarity of approximately 35–45% with known conserved domains for the CBM32 family or representative members. This multifunctional CBM was genetically engineered as a fusion partner with various target enzymes, and its efficacy in facilitating one-step purification and immobilization on diverse polysaccharide-based microspheres was systematically evaluated. Furthermore, the operational stability and catalytic performance of the immobilized enzymes were thoroughly investigated through repeated reaction studies. This innovative approach offers an eco-friendly and cost-efficient alternative to conventional immobilization methods, with significant potential in bio-manufacturing.

## 2. Materials and Methods

### 2.1. Chemicals, Strains, Plasmids, and Genes

Chitosan, chitin, cellulose, L-lysine hydrochloride, and L-glutamate sodium were purchased from Aladdin Reagent Co., Ltd. (Shanghai, China). All other chemicals utilized in this study were of analytical purity and were procured from local suppliers, unless otherwise indicated. A plasmid mini-lift kit and DNA gel recovery kit were obtained from Tiangen Co., Ltd. (Shanghai, China), and the restriction enzymes were purchased from TaKaRa Bio Co., Ltd. (Dalian, China). The high-fidelity DNA polymerase and ClonExpress Ultra One Step Cloning Kit V2 were purchased from Vazyme Biotech Co., Ltd. (Nanjing, China). CBM genes from *Paenibacillus* sp. IK-5 and primers were synthesized by General Bio Co., Ltd. (Chuzhou, China). The bacterial strains *Escherichia coli* DH5α and BL21 (DE3), utilized for molecular cloning and recombinant protein expression, respectively, were commercially sourced from TransGen Biotech Co., Ltd. (Beijing, China). Carbohydrate-binding modules, L-lysine decarboxylase (CadA), L-glutamic acid oxidase (LGOX), formate dehydrogenase (*Cb*FDH), green fluorescent protein (EGFP), yellow fluorescent protein (YFP), and red fluorescent protein (mCherry) genes were preserved in our laboratory.

### 2.2. Gene Cloning and Expression

The schematic drawing of recombinant plasmid construction is shown in Figure S1, and the related specific primers are listed in Table S1. The recombinant plasmid pET28a-*CadA-CBM* was constructed by inserting the amplified *CadA* gene into the *BamH*I/*Nde*I-linearized pET28a-*CBM* vector through seamless cloning using a One Step Cloning Kit. Subsequently, the enhanced green fluorescent protein (*EGFP*) gene was fused to the N-terminus of the *CadA* gene, thereby generating the recombinant plasmid pET28a-*EGFP-CadA-CBM*. The recombinant plasmid pET28a-*CBM-LGOX* was generated by inserting the PCR-amplified *LGOX* gene into the *Hind*III/*Xho*I-linearized pET28a-*CBM* vector via seamless cloning. To enable fluorescence-based tracking, the red reporter gene (*mCherry*) was translationally fused to the N-terminus of the *CBM* via overlap extension PCR, yielding the final construct pET28a-*mCherry-CBM-LGOX*. The recombinant plasmid pET28a-*CBM-CbFDH* was generated by inserting the PCR-amplified *CbFDH* gene into the *Hind*III/*Xho*I-linearized pET28a-*CBM* vector via seamless cloning. To enable fluorescent tagging, the yellow fluorescent protein gene (*YFP*) was translationally fused to the N-terminus of the *CBM*, yielding the final construct pET28a-*YFP-CBM-CbFDH*. All plasmids were transformed into *E. coli* DH5α competent cells and validated by sequencing.

For gene expression, the recombinant plasmids containing confirmed genes were transformed into *E. coli* BL21 (DE3), incubated in an LB liquid medium containing 50 μg/mL of kanamycin, and then cultured at 37 °C with shaking at 200 rpm. When the optical density (OD_600_) of the culture was about 0.6–0.9, isopropyl β-D-thiogalactoside (IPTG) was added to a final concentration of 1 mM for protein induction, and the culture was incubated at 18 °C for 16–18 h.

Cultures were collected by centrifugation at 6000× *g* and 4 °C for 10 min, after which the pellet was gently resuspended in 50 mM phosphate-buffered saline (pH 7.0) and then lysed using JY92-IIN ultrasonication (Ningbo Xinzhi Biotechnology, Ltd., Ningbo, China). The lysate was centrifuged at 8000× *g* for 15 min, and the supernatant was collected as the crude enzyme and stored at 4 °C before use.

### 2.3. Preparation of Chitin, Chitosan, and Cellulose Microspheres

Chitin, chitosan, and cellulose microspheres were prepared according to a previous study with minor adjustments [23]. Taking chitosan microspheres (CMs) as an example, the manufactured chitosan microspheres were designated as CM-X, where X represented the different chitosan content in CM samples. For instance, CM-10 denotes microspheres prepared using a chitosan/isooctane solution with a 10:90 mass ratio. CM-12.5, CM-20, and CM-33 were prepared using an identical protocol to CM-10, with adjusted chitosan/isooctane solution mass ratios.

For 10% chitosan microspheres, the procedure is as follows: Chitosan powder (1 g) was dissolved in 39 g of a LiOH/KOH/urea/H_2_O solution (8:7:8:77 mass ratio) with three freeze/thaw cycles [24]. Subsequently, 10 g of the fully dissolved chitosan solution was transferred to a pre-cooled oil phase containing 90 g of isooctane and 6 g of Span 85. The suspension was first agitated (1000 rpm, 1 h) under cryogenic conditions and then solidified by heating and stirring at 80 °C for 10 min. Finally, chitosan microspheres were harvested via centrifugation, followed by three sequential washes with 95% ethanol (an ethanol-to-microsphere ratio of 5:1, *v*/*v*) and deionized water (a water-to-microsphere ratio of 10:1, *v*/*v*) to purge residual isooctane.

For 10% chitin microspheres, the procedure is as follows: First, 1.25 g of chitin was dissolved in 23.75 g of a NaOH/urea/H_2_O (11:4:85) solution with three freeze/thaw cycles. Subsequently, 10 g of the completely dissolved chitin solution was transferred to a pre-cooled oil phase (90 g of isooctane, 2.2 g of Span 85, and 0.3 g of Tween 85). The resulting suspension was agitated (1000 rpm, 1 h) under cryogenic conditions and solidified by heating and stirring at 60 °C for 10 min. It was then adjusted to neutral using 10% HCl. Finally, the chitin microspheres were collected and washed following the same protocol as described previously.

For 10% cellulose microspheres, the procedure is as follows: First, 1 g of cellulose powder was dissolved in 24 g of a NaOH/urea/H_2_O (7:12:81) solution under continuous stirring at −80 °C until complete dissolution. Subsequently, 10 g of the cellulose solution was added to a pre-cooled oil phase (90 g of isooctane and 6 g of Span 85). Next, 4 g of isooctane and 0.3 g of Tween 85 were incorporated into the mixture. The resulting suspension was stirred at 1000 rpm for 1 h under cryogenic conditions and then adjusted to neutral. Finally, the cellulose microspheres were collected and washed following the same protocol as described previously.

### 2.4. Simultaneous Purification and Immobilization of Enzymes Using CMs

The prepared CMs were used as a carrier for enzyme immobilization. To conduct the immobilization process, 0.01 g of CMs was incubated with different fusion enzymes (1.0 g/L of CadA-CBM, CBM-LGOX, and CBM-*Cb*FDH) in Eppendorf tubes. The incubation temperature was maintained at 25 °C, and the mixture was shaken at 200 rpm for a duration of one hour. The immobilized enzyme was collected via a process of centrifugation. Thereafter, the residual proteins were eliminated through a series of washes with 1 M NaCl. The quantity of adsorbed protein was calculated in accordance with Equation (1), with the protein concentration being ascertained using the Bradford method [25].

(1) [Immobilized protein] = Protein concentration1 − Protein concentration2

Protein concentrations 1 and 2 are indicative of the protein concentrations present in the supernatant prior to and subsequent to immobilization, respectively.

### 2.5. Characterization of the Immobilized Enzymes

Prior to the characterization process, the samples underwent a freeze-drying process under vacuum.

Fourier-transform infrared spectroscopy (FT-IR) spectral profiles of CM, CadA-CBM, and the CM@CadA-CBM composite were recorded on a Nicolet iS5 spectrometer (Thermo Fisher Scientific, Waltham, MA, USA) operating in an attenuated total reflectance (ATR) mode. The analytical specimens were fabricated as potassium bromide (KBr) pellets for infrared spectroscopic characterization.

The morphology of microspheres and immobilized enzymes was observed using a scanning electron microscope (Hitachi Regulus 8100, Tokyo, Japan) at an accelerating voltage of 10 kV, after being coated with gold.

The investigation of CM@EGFP-CadA-CBM was performed using a fluorescence microscope (Olympus BX51, Evident Corporation, Tokyo, Japan).

The elemental analyses (EA) of CM, CadA-CBM, and CM@CadA-CBM were determined using a Vario EL Cube instrument (Elementar Analysensysteme GmbH, Langenselbold, Germany).

A thermal gravimetric analysis (TGA) was conducted using a NETZSCH TG 209 F1 Libra thermogravimetric analyzer. The heating rate was 10 °C/min from 30 °C to 800 °C under nitrogen atmospheres (100 mL/min).

Surface area analysis and porosity characterization were conducted through N_2_ physisorption measurements at liquid nitrogen temperature (77 K) using an Autosorb iQ MP analyzer (Quantachrome Instruments, Boynton Beach, FL, USA). Surface area calculations employed the Brunauer–Emmett–Teller [26] method, while pore size distribution profiles were derived from Barrett–Joyner–Halenda (BJH) and nonlocal density functional theory (NLDFT) models using built-in analytical modules.

### 2.6. Enzyme Activity Assays

CadA-CBM activity: The reaction mixture containing CadA-CBM, 25 mM L-lysine, and 0.1 mM pyridoxal phosphate was prepared in a phosphate-citrate buffer (pH 6.2) and thermostated at 45 °C for an incubation period of 10 min. One unit of enzyme activity (U) was designated as the quantity of CadA-CBM necessary to catalyze the consumption of 1 μmol of L-lysine per minute under standardized conditions (45 °C, pH 6.2).

CBM-LGOX activity: CBM-LGOX was added to a phosphate-citrate buffer (pH 7.2) supplemented with 25 mM glutamate, followed by incubation at 37 °C for 10 min. One unit of enzyme activity (U) was designated as the quantity of CBM-LGOX necessary to catalyze the conversion of L-glutamate to 1 μmol of α-ketoglutarate at 37 °C and at a pH of 7.2 per minute.

CBM-*Cb*FDH activity: CBM-*Cb*FDH was added to a phosphate-buffered saline (50 mM, pH 7.0) containing 25 mM formic acid and 0.1 mM NAD. After 15 min of incubation at 37 °C, NADH production was quantified by monitoring absorbance at 340 nm. The activity of one unit of enzyme was defined as the amount of enzyme required to generate 1 μmol of NADH per minute.

The SBA-40E biosensing analyzer (Shandong Academy of Sciences Institute of Biology, Ji’nan, China) was employed to determine the concentrations of L-lysine and L-glutamic acid.

A UV spectrophotometer was used to measure the absorbance changes in NADH at 340 nm.

### 2.7. Effect of Temperature and pH on Enzyme Activity

The effect of temperature and pH on enzyme activity was determined by measuring the enzyme activity of CadA-CBM and CM@CadA-CBM at different temperatures (25–65 °C) and pH levels (5.0–8.0). To determine thermal stability, enzymes without substrates were incubated in an acidic 100 mM disodium hydrogen phosphate buffer (pH 6.2) at 25–65 °C for 6 h, and the residual activities were determined. For pH stability, the enzymes without substrate were incubated at pH 5.0–8.0 for 6 h, and the residual activities were determined.

### 2.8. The Reusability of the Immobilization Enzymes

To assess the operational stability of the immobilized enzyme, catalyst recovery was performed after each reaction cycle through centrifugation (8000× *g*, 4 °C) followed by reintroduction into a new reaction system. The reaction temperature was identified as the optimal temperature for each enzyme, and the reaction system was referenced to the enzyme activity assay described above. This recycling procedure was systematically repeated for ten consecutive batches to evaluate the biocatalyst’s reusability.

### 2.9. Analytical Methods

All recombinant protein samples were analyzed by reductive sodium dodecyl sulfate–polyacrylamide gel electrophoresis (SDS-PAGE) with a 20 mM 2-mercaptoethanol incubation. A premixed protein marker (Takara Biotechnology Co., Ltd., Nanjing, China) containing 180, 140, 100, 80, 60, and 45 kDa protein bands was used as the molecular mass standard.

## 3. Results and Discussion

### 3.1. Fusion of Carbohydrate-Binding Module and Target Proteins

**Figure 1:**
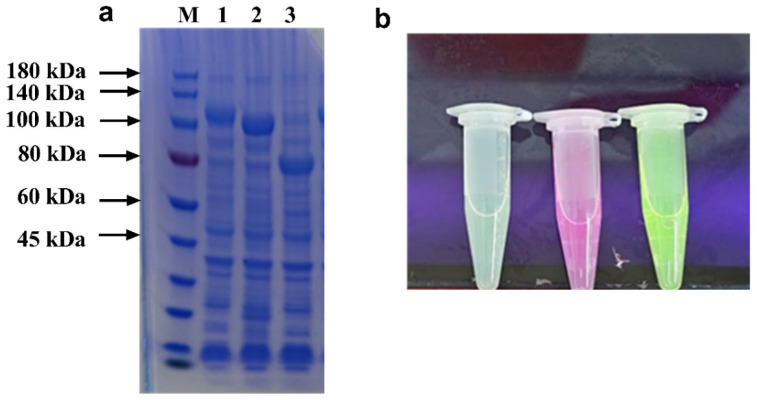
(**a**) SDS-PAGE analysis of expression of CBM fusion protein. Lane M: protein marker; lanes 1–3: supernatants of CadA-CBM, CBM-LGOX, and CBM-*Cb*FDH. (**b**) Photographs of three fluorescent protein-labeled enzymes under UV light.

The inherent substrate recognition specificity and binding affinity of carbohydrate-binding modules (CBMs) establish them as highly efficient tools for both enzyme purification and immobilization processes [19,27]. In this study, CBMs derived from five distinct sources were comparatively analyzed, revealing that the majority demonstrate monospecific binding characteristics with high affinity toward individual substrates (Table 1). Notably, prior studies have identified and characterized several CBMs exhibiting diverse binding capabilities, consequently broadening the spectrum of potential immobilization matrices for biocatalytic applications. A representative example is demonstrated by Hanazono et al., who reported a chitinase enzyme from *T. kodakarensis* composed of two catalytic domains and three binding domains (ChBD1, ChBD2, and ChBD3). Experimental evidence has confirmed that ChBD2 and ChBD3 exhibit dual-substrate specificity, demonstrating significant binding affinity toward both chitin and cellulose [28]. To assess the multifunctional potential of the chitosan-binding module (CBM) derived from *Paenibacilus* sp. IK-5 for concurrent enzyme purification and immobilization on diverse polysaccharide matrices. This module is part of the CBM32 family and is located in the C-terminal region of IK-5 chitosanase. It contains two identical carbohydrate-binding modules (DD1 and DD2) that share the same structure. Polysaccharides generally bind to DD2 [29]. Three distinct target enzymes—L-lysine decarboxylase (CadA), L-glutamate oxidase (LGOX), and formate dehydrogenase (*Cb*FDH)—were chosen as test enzymes in the experiment based on their more convenient conditions for detection. The expression of the recombinant fusion proteins was first investigated. As demonstrated in Figure 1a, CadA-CBM exhibited a distinct band within the range of 100–140 kDa, CBM-LGOX demonstrated a clear band around 100.0 kDa, and CBM-CbFDH exhibited a clear band around 80 kDa, which corresponded to their theoretical molecular weights of 110.2 kDa, 97.8 kDa, and 68.4 kDa, respectively. This result indicates the successful expression of the CBM fusion protein. To facilitate the convenient visualization and detection of immobilized enzymes, EGFP/mCherry/YFP were employed as reporter signals. The presence of green, red, and yellow fluorescence was observed in EGFP-CadA-CBM, mCherry-CBM-LGOX, and YFP-CBM-CbFDH, respectively, under UV–visible spectrophotometer analysis. This finding serves to confirm the successful expression of fusion enzymes (Figure 1b).

### 3.2. Preparation and Characterization of Three Polysaccharide Microspheres

**Figure 2:**
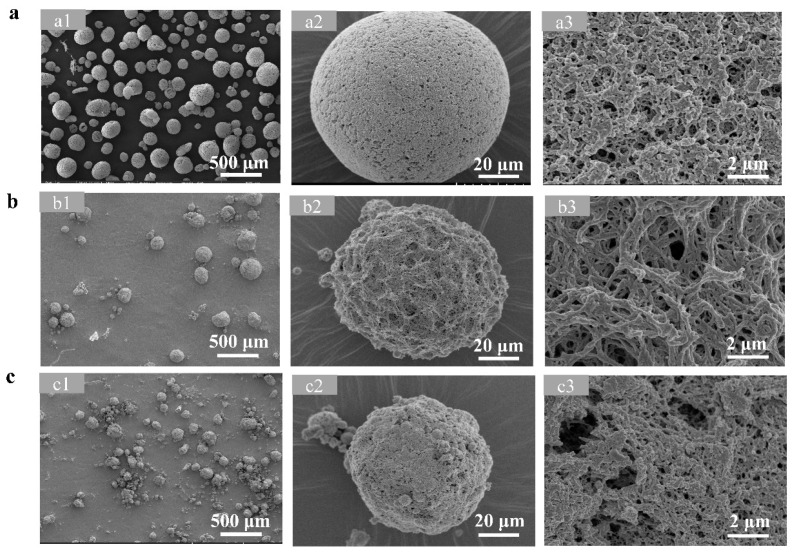
SEM images of chitosan (**a**), chitin (**b**), and cellulose (**c**) microspheres.

Chitosan, chitin, and cellulose microspheres were successfully prepared using an emulsification method. The morphology of three microspheres was characterized using scanning electron microscopy (SEM). As demonstrated in Figure 2, all types of microspheres exhibited a regular spherical and porous structure, which is consistent with previous reports that their porous structure may facilitate the subsequent immobilization of enzyme proteins [33,34,35].

### 3.3. Simultaneous Purification and Immobilization of Fusion Protein Based on Three Microspheres

**Figure 3:**
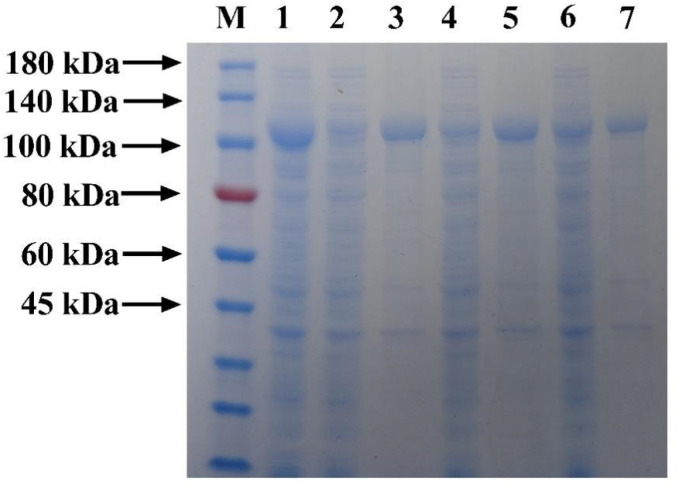
SDS-PAGE analysis of CadA-CBM immobilization on three types of microspheres. Lane M: protein marker; lane 1: CadA-CBM crude enzyme; lanes 2, 4, and 6: supernatants after immobilization using chitosan microsphere, chitin microsphere, and cellulose microsphere; and lanes 3, 5, and 7: eluted solutions of chitosan microsphere, chitin microsphere, and cellulose microsphere with immobilized enzymes.

CadA-CBM was used as a model enzyme to investigate the simultaneous purification and immobilization process on three polysaccharide microspheres. The adsorption of CadA-CBM on the three microspheres was investigated by SDS-PAGE. The presence of crude enzyme bands of CadA-CBM could be observed in lane 1; the single elution band in lanes 3, 5, and 7 at 100 kDa indicated the effective adsorption of CBM on three microspheres (Figure 3). This finding demonstrated the broad applicability of the CBM for polysaccharide-based immobilization platforms.

**Figure 4:**
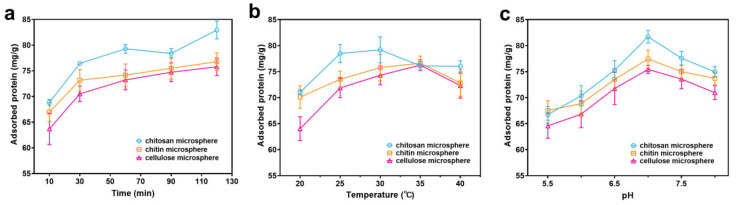
The effects of time (**a**), temperature (**b**), and pH (**c**) on protein adsorption.

**Figure 5:**
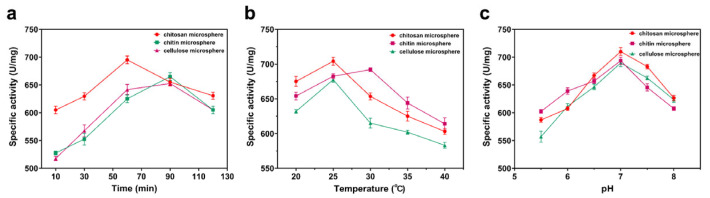
The effects of time (**a**), temperature (**b**), and pH (**c**) on specific activity.

Furthermore, the protein adsorption capacity (Figure 4) and specific activity (Figure 5) of the three microspheres were investigated. The loading of the microspheres reached equilibrium at 60 min, although prolonging the time would increase the protein adsorption, but at the same time, the enzyme activity would be reduced. The optimal loading and specific enzyme activity were demonstrated at 25 °C and at a pH of 7.0. Thus, the optimum adsorption conditions were 60 min, 25 °C, and pH 7.0. Chitosan microspheres demonstrated significantly higher protein loading compared to chitin and cellulose microspheres. The immobilization rates of the three types of microspheres for fusion proteins with CBM were investigated under optimal adsorption conditions. The findings demonstrated that, in comparison with chitin and cellulose microspheres, the immobilization rate of the chitosan microspheres was elevated, reaching 82.25 ± 2.66% (Table S3). Consequently, chitosan microspheres were chosen for subsequent experimentation.

### 3.4. The Specific Surface Areas of Chitosan Microspheres on Protein Adsorption Capacity

**Figure 6:**
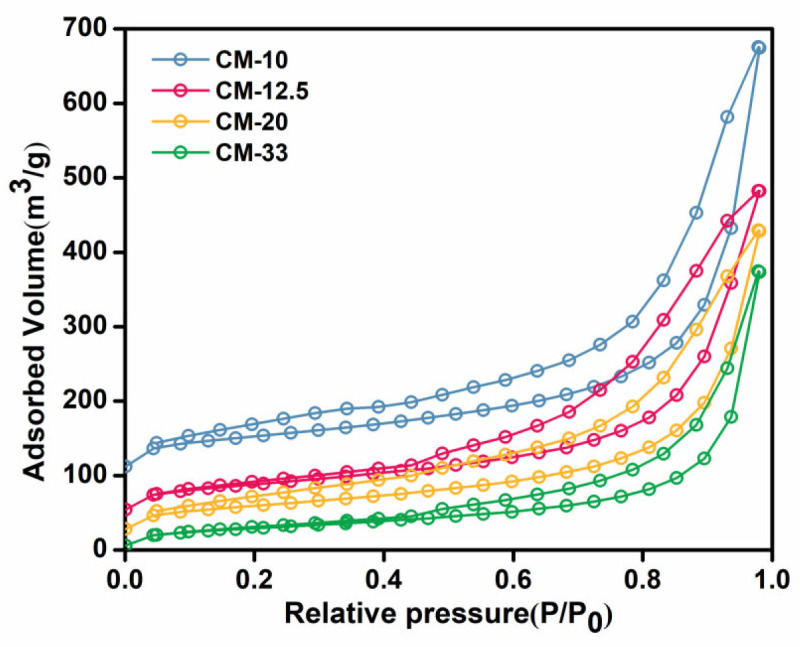
N_2_ adsorption and desorption isotherms of CM-10, CM-12.5, CM-20, and CM-33.

The enzyme loading capacity represents a crucial performance metric for assessing carrier efficacy in immobilization processes, demonstrating a direct correlation with the carrier’s specific surface area (SSA) and available binding sites. Thus, the fabrication of several chitosan microspheres with varying SSAs was carried out. As demonstrated in Figure 6, the N_2_ adsorption/desorption isotherms of CM samples indicate that the SSAs of CM-10, CM-12.5, CM-20, and CM-33 were 192.05, 160.65, 145.91, and 107.01 m^2^/g, respectively (Table S2). This finding suggests a substantial enhancement in the SSAs of CMs in comparison with those of the chitosan powder. Furthermore, a notable increase in the total pore volume of the CMs was observed as the chitosan solution concentration decreased from CM-33 to CM-10. This finding indicates that the SSAs of microspheres correlate with the chitosan content used during synthesis. In subsequent experiments, chitosan microspheres with varying specific surface areas were compared, and the one exhibiting optimal performance was selected for further experiments.

**Figure 7:**
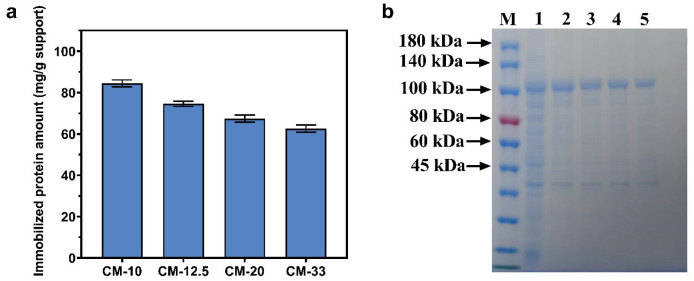
(**a**) The maximum protein loading ability of different carriers. (**b**) SDS-PAGE analysis of CadA-CBM immobilized on chitosan microspheres with different parameters. Lane M, protein marker; lane 1, CadA-CBM crude enzyme; lane 2, CadA-CBM eluted after immobilization using CM-10; lane 3, CadA-CBM eluted after immobilization using CM-12.5; lane 4, CadA-CBM eluted after immobilization using CM-20; lane 5, CadA-CBM eluted after immobilization using CM-33.

The protein adsorption capacity of CMs with varying specific surface areas was quantitatively evaluated under controlled experimental conditions (Figure 7a). Among the prepared microspheres, CM-10 demonstrated a superior adsorption capacity (82.5 ± 2.5 mg/g), which may be attributed to its significantly enhanced SSA, thereby providing an increased density of accessible binding sites for CBM fusion proteins. The observed notable decrease in the loading capacity of CM-20 and CM-33 primarily stems from their diminished SSAs, which directly correlate with a decreased availability of specific binding sites, ultimately leading to a lower immobilization efficiency. SDS-PAGE analysis confirmed successful CadA-CBM adsorption across carriers (Figure 7b): lane 1 displayed a distinct overexpression band at 100 kDa, while lanes 2–5 exhibited relatively single eluted bands at the same molecular weight. Furthermore, the eluted protein quantities in lanes 2–5 align with the adsorption trends observed in Figure 7b, validating the consistency of the experimental results. Consequently, CM-10 was selected for further experiments.

### 3.5. Characterization of Enzyme Immobilization on Chitosan Microspheres

**Figure 8:**
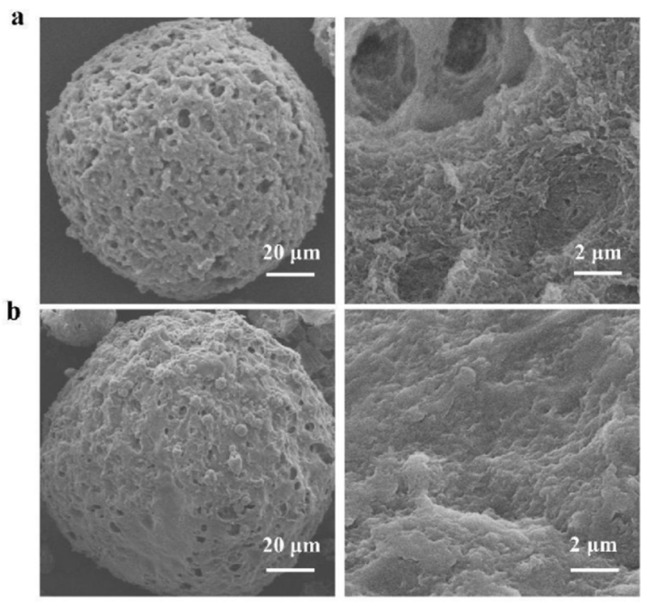
SEM images of chitosan microspheres before (**a**) and after (**b**) enzyme immobilization.

Scanning electron microscopy was conducted to characterize the CM before and after enzyme immobilization (Figure 8). SEM analysis revealed that the CM displayed a characteristic fibrous and porous structure prior to immobilization. Following this process, it was possible to discern the presence of distinct protein aggregates on the microsphere matrices post-immobilization, thus confirming the successful immobilization of the enzyme. This finding correlates with previous reports [36,37].

**Figure 9:**
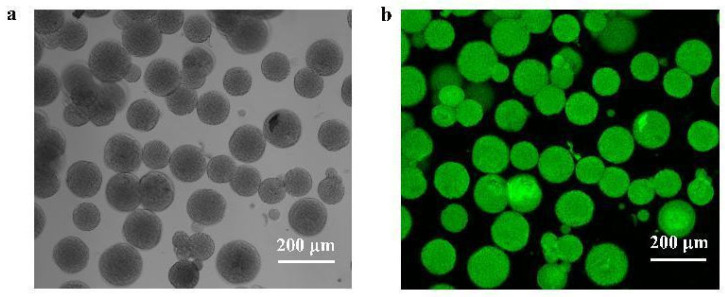
Fluorescence microscopy images of CadA-CBM immobilization on CMs: (**a**) bright field and (**b**) fluorescence field.

The spatial distribution of the CadA-CBM on CM was visualized by means of fluorescence microscopy (Figure 9). Bright-field imaging revealed well-defined spherical morphologies of CM@CadA-CBM, while fluorescent imaging displayed green fluorescent signals from EGFP-CadA-CBM, indicating successful enzyme immobilization.

**Figure 10:**
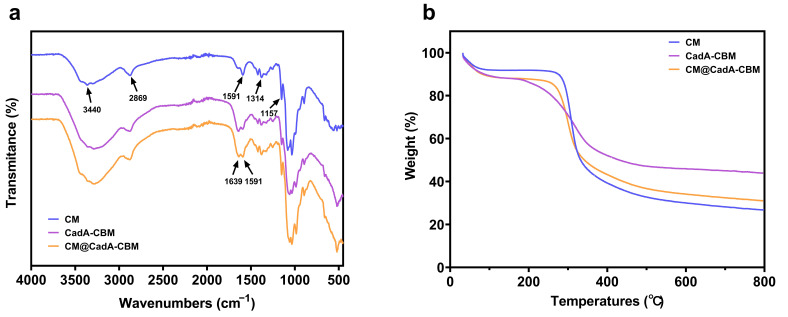
(**a**) FT-IR spectra of chitosan microspheres (blue line), CadA-CBM (purple line), and CM@CadA-CBM (orange line). (**b**) TGA curves of chitosan microspheres (blue line), CadA-CBM (purple line), and CM@CadA-CBM (orange line).

The chemical structures of CMs, CadA-CBM, and CM@CadA-CBM were investigated by Fourier-transform infrared spectroscopy. As demonstrated in Figure 10a, the distinctive absorption bands of chitosan were 3440 cm^−1^ (N-H stretching), 2869 cm^−1^ (-CH_2_ asymmetric stretching), 1591 cm^−1^ (-NH bending of amide II), and 1314 cm^−1^ (-CN stretching of amide III), as well as 1157 cm^−1^ for C-O stretching vibration [38,39,40]. CadA-CBM possessed characteristic peaks at 1591 cm^−1^ and 1639 cm^−1^, which correspond to the stretching vibration of the protein-specific amide II bond (-NH-) and amide II bond (C=O), respectively [41]. After immobilization, the peaks of CadA-CBM (1591 cm^−1^ and 1639 cm^−1^) covered the peak of chitosan (1591 cm^−1^), also demonstrating that CadA-CBM has been immobilized on the CMs.

The thermal stability profiles of CMs, CadA-CBM, and CM@CadA-CBM were analyzed through TGA (Figure 10b). A comparative analysis revealed that while the thermal degradation pattern of CM@CadA-CBM differed significantly from that of pure chitosan, it exhibited remarkable similarity to the thermal behavior of the free CadA-CBM fusion protein. The thermogravimetric profile of CM revealed an initial mass loss of approximately 10% within the temperature range of 30–110 °C, which could be attributed to the evaporation of physically adsorbed water molecules from the polysaccharide matrix [42]. However, the majority of mass loss occurred within the range 280–400 °C, resulting in the formation of carbonaceous residues [43]. For both CadA-CBM and CM@CadA-CBM, an initial mass reduction of approximately 17 wt% was observed between 30 and 200 °C because of the removal of water. Subsequently, a significant decomposition phase was detected between 200 and 270 °C, with CadA-CBM and CM@CadA-CBM showing comparable mass (7 wt%), which could be attributed to the breakdown of the enzyme molecules.

Elemental analyses were performed to determine the C, H, and N contents of CMs, CadA-CBM, and CM@CadA-CBM (Table S3). The analytical results revealed that the C, H, and N contents of CM@CadA-CBM were 36.33%, 6.26%, and 7.10%, respectively, which were intermediate values between those of CMs and CadA-CBM. These results also provided conclusive evidence for the effective immobilization of the CadA-CBM on CMs.

### 3.6. Enzymatic Properties of Immobilized Enzymes

**Figure 11:**
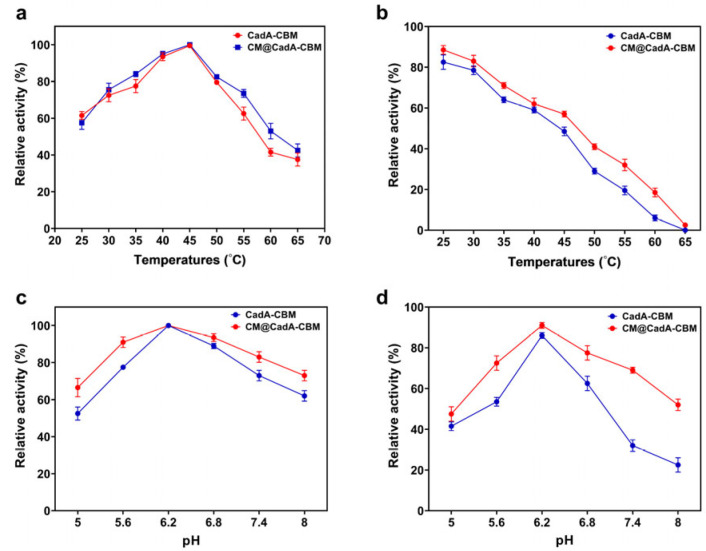
(**a**) The optimum temperature for CadA-CBM and CM@CadA-CBM; (**b**) the temperature stability for CadA-CBM and CM@CadA-CBM; (**c**) the optimum pH for CadA-CBM and CM@CadA-CBM; (**d**) the pH stability for CadA-CBM and CM@CadA-CBM.

The effect of temperature on enzyme activity was investigated. The optimal temperature for CadA-CBM and CM@CadA-CBM was 45 °C (Figure 11a). As shown in Figure 11b, the temperature stability of CadA-CBM and CM@CadA-CBM was explored, and CM@CadA-CBM showed higher temperature stability. An investigation was conducted into the effects of pH on the activities of CadA-CBM and CM@CadA-CBM. The maximum activity of CadA-CBM and CM@CadA-CBM was achieved at pH 6.2; more than 60% of the highest activity can be obtained at pH 8.0 (Figure 11c). As shown in Figure 11d, the pH stability of CadA-CBM and CM@CadA-CBM was evaluated. The results demonstrate that CadA-CBM exhibits enhanced pH stability following immobilization, retaining over 50% of its initial enzymatic activity at pH 8.0.

### 3.7. Reusability of Immobilized Enzymes

**Figure 12:**
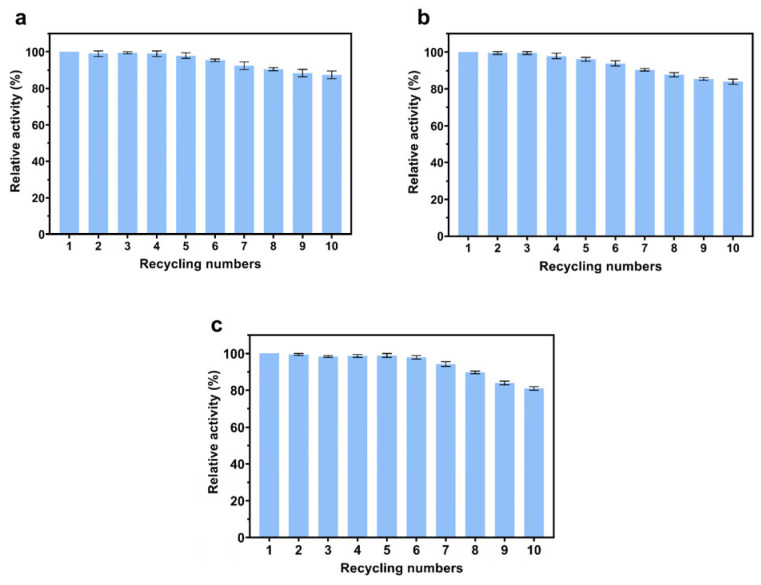
The reusability of three immobilized enzymes: (**a**) CM@CadA-CBM, (**b**) CM@CBM-LGOX, and (**c**) CM@CBM-*Cb*FDH.

The operational stability and reusability of immobilized enzymes are crucial for their successful implementation in industrial-scale biotransformation processes. To assess the reusability of immobilized enzyme systems, three different enzyme constructs were selected for comprehensive analysis. As illustrated in Figure 12, all immobilized enzyme preparations (CM@CadA-CBM, CM@CBM-LGOX, and CM@CBM-*Cb*FDH) maintained exceptional catalytic activity, retaining >80% of their initial activity after ten operational cycles. These results demonstrated the broad applicability of the CBM-based immobilization strategy, effectively addressing the inherent limitations of conventional free-enzyme systems, particularly their economic constraints and limited reusability.

## 4. Conclusions

This study successfully developed an innovative integrated platform for simultaneous purification and immobilization through CBM fusion technology. The engineered CBM fusion enzymes demonstrated efficient immobilization onto polysaccharides through hydrophobic interactions and ionic forces. The resulting immobilized biocatalysts exhibited high catalytic performance and remarkable operational stability, maintaining significant activity through multiple reuse cycles. However, a decrease in the activity of the target enzyme was observed after CBM fusion, which may be due to inappropriate fusion sites or linker peptides affecting the enzyme folding process. A rational design of the fusion site or the systematic optimization of linker peptide lengths/sequences could minimize folding interference by preserving the enzyme’s conformational flexibility. This novel approach proposes a significant advancement in enzyme immobilization technology by eliminating the resource-intensive purification steps inherent in conventional methods, thereby offering a promising approach for enzyme immobilization in industrial biocatalytic applications.

## Figures and Tables

**Table 1:** Substrate specificity of five different sources of CBMs.

	Strains	CAZyme Source	Ligands	Sequence ID	References
1	*Chitinibacter* sp. GC72	Chitinase	Chitin	WP_157670862.1	[22]
2	*Chitinolyticbacter meiyuanensis*	Chitinase	Chitin	WP_148716959.1	[30]
3	*Cellulomonas fimi*	β-1,4-glucanase	Cellulose	AAB34464.1	[31]
4	*Trichoderma reesei*	Cellobiohydrolase I	Cellulose	1CBH_A	[32]
5	*Paenibacillus* sp	Chitosanase	Chitosan	BAB64835.1	[29]

## Data Availability

The original contributions presented in the study are included in the article/Supplementary Material, further inquiries can be directed to the corresponding authors.

## Supplementary Materials

The following supporting information can be downloaded at: https://www.mdpi.com/article/10.3390/foods14111981/s1. Figure S1. Schematic diagrams depicting plasmid construction. Table S1. Primers used in this work. Table S2. The SSA parameters of CM-10, CM-12.5, CM-20 and CM-33. Table S3. Immobilization rates of three microspheres under optimal adsorption conditions. Table S4. Elemental analysis of chitin before and after enzyme immobilization.
