# Supplementary material for: A Chitosan-Binding Protein Mediated the Affinity Immobilization of Enzymes on Various Polysaccharide Microspheres
Source: Foods. 2025 Jun 4;14(11):1981. doi: 10.3390/foods14111981 (PMC12154041; doi:10.3390/foods14111981)
Supplement: Supplementary file 1 [file foods-14-01981-s001.zip › foods-3647258-supplementary.pdf]

Support information

A chitosan binding protein mediated affinity immobilization of enzymes on various polysaccharide microspheres

Dexin Zhao, Shiguo Peng, Feifei Chen, Alei Zhang * and Kequan Chen *

State Key Laboratory of Materials-Oriented Chemical Engineering, College of Biotechnology and Pharmaceutical Engineering, Nanjing Tech University, Nanjing, 211816, China.

*Corresponding author.

E-mail address: zhangalei@njtech.edu.cn; kqchen@njtech.edu.cn

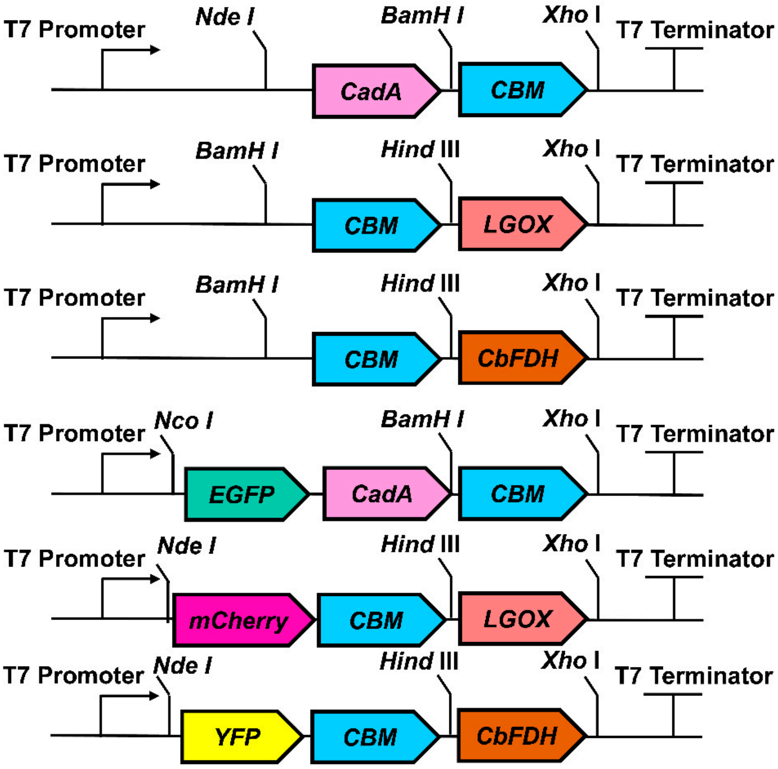

Figure S1. Schematic diagrams depicting plasmid construction.

Table S1. Primers used in this work.

Primers	Sequence (5'-3')
CadA-F	CTGGTGCCGCGCGGCAGCCATATGAACGTTATTGCAATATTGAATCAC
CadA-R	ATTCAGGGCCAGGTTGGATCCTTTTTTGCTTTCTTCTTTCAATACCTT
LGOX-F	GAGGTGTATGGTAAGCTTGCGATGAACGTTATTGCAATATTGAATCAC
LGOX-R	GTGGTGGTGCTCGAGTTTTTTGCTTTCTTCTTTCAATACCTT
CbFDH-F	TGGGAATTTGAGGTGTATGGTAAGCTTATGAAGATCGTTTTAGTCTTATATGATGCTGGTAAG
CbFDH-R	GTGGTGGTGGTGCTCGAGTTTCTTATCGTGTTTACCGTAAGCTTTAGTAAC
EGFP-F	ACTTTAAGAAGGAGATATACCATGGTGAGCAAGGGCGAG
EGFP-R	GATTCAATATTGCAATAACGTTCATATGCTTGTACAGCTCGTCCATGCC
mCherry-F	CCGCGCGGCAGCCATATGGTTAGCAAAGGTGAAGAAGATAATATG
mCherry-R	CAGGGCCAGGTTGGATCCGGTACTATGGCGGCCTTC
YFP-F	CCGCGCGGCAGCCATATGATGGTGAGCAAGGGCGAG
YFP-R	CAGGGCCAGGTTGGATCCCTTGTACAGCTCGTCCATGCC

Table S2. The SSA parameters of CM-10, CM-12.5, CM-20 and CM-33.

Samples	Mean diameter (μm)	S_{BET}[a] (m^2/g)	V_{total}[b] (cm^3/g)
CM-10	73.03	192.05	0.89
CM-12.5	78.26	160.65	0.68
CM-20	78.99	145.91	0.63
CM-33	95.40	107.01	0.58

a S_{BET} is specific surface area.

b Vtotal is total pore volume.

Table S3. Immobilization rates of three microspheres under optimal adsorption conditions.

Samples	Immobilization rates (%)
chitosan microspheres	82.25±2.66
chitin microspheres	76.54±2.98
cellulose microspheres	75.41±2.89

Table S4. Elemental analysis of chitin before and after enzyme immobilization.

Sample name	Elemental content (%)		
	C	H	N
CM	41.34	6.48	7.74
CadA-CBM	29.92	5.09	9.14
CM@CadA-CBM	36.33	6.26	8.10
